# The Effects of 3D Immersion Technology (3Scape) on Mental Health in Outpatients From a Short-Term Assessment, Rehabilitation, and Treatment Program: Feasibility Protocol for a Randomized Controlled Trial

**DOI:** 10.2196/25017

**Published:** 2021-09-14

**Authors:** Antonio Miguel-Cruz, Anna-Maria Ladurner, Megan Kohls-Wiebe, David Rawani

**Affiliations:** 1 University of Alberta Edmonton, AB Canada; 2 Glenrose Rehabilitation Research Innovation and Technology Edmonton, AB Canada; 3 Glenrose Rehabilitation Hospital Edmonton, AB Canada

**Keywords:** technology assessment, mental health, technology for rehabilitation, clinical engineering, biomedical engineering

## Abstract

**Background:**

Mental health conditions are prevalent among Canadians and are a leading cause of disability. Each year, 1 in 5 Canadians experiences a mental health issue. A total of 5% of people aged ≥65 years perceive their mental health as fair or poor, and 6.3% of them have mood disorders. Regarding older adults with cognitive impairments such as dementia, up to 40%-50% of them experience depression at some point. We believe that older adults can benefit significantly from information and telecommunication technologies as a strategy for improving mental health conditions such as depression and anxiety, while simultaneously improving their quality of life. 3Scape Systems Inc is an Alberta-based private company that has produced a series of specialized 3D videos designed to simulate real-life events and engage individuals living with mental health disorders and cognitive impairments such as dementia.

**Objective:**

This study aims to explore the trial design and effects of 3Scape videos on older adults’ symptoms of depression and anxiety and the efficacy of this technology in improving the quality of life of patients attending the Short-Term Assessment, Rehabilitation, and Treatment Psychiatry Day Hospital program at Glenrose Rehabilitation Hospital and to provide data to estimate the parameters required to design a definitive randomized controlled trial.

**Methods:**

The trial will use a randomized controlled design comprising 15 intervention participants and 15 control group participants. The participants will be adults aged ≥65 years who are cognitively intact or have minimal cognitive impairment (ie, Montreal Cognitive Assessment score ≥18), and are clients of the Short-Term Assessment, Rehabilitation, and Treatment Psychiatry Day Hospital program at Glenrose Rehabilitation Hospital. This study’s primary outcome variables are related to clients’ depressive and anxiety symptoms and their quality of life. The control group will receive the standard of care (ie, the Short-Term Assessment, Rehabilitation, and Treatment Psychiatry Day Hospital program at Glenrose Rehabilitation Hospital). The intervention group will receive the same standard of care as the control group and will use 3Scape Systems videos for therapeutic activities.

**Results:**

Our study is currently on hold because of the COVID-19 pandemic. The recruitment process is expected to resume by November 2021, and the primary impact analysis is expected to be conducted by February 2022.

**Conclusions:**

This study will provide valuable information such as the measurement of comparative intervention effects, perception of older adults and mental health therapists about the 3Scape Systems, the associated costs of treatment, and product costs. This will contribute to the evidence planning process, which will be crucial for the future adoption of 3Scape Systems.

**Trial Registration:**

International Standard Randomized Controlled Trial Number (ISRCTN): 93685907; https://www.isrctn.com/ISRCTN93685907.

**International Registered Report Identifier (IRRID):**

PRR1-10.2196/25017

## Introduction

### Background and Rationale

Mental health disorders disrupt a person’s thinking, feeling, mood, and ability to relate to others, affecting their daily functioning. Mental health conditions are prevalent among Canadians. Mental health conditions are the leading causes of disability, with 1 in 5 Canadians experiencing a mental health concern each year. In Canada, mental health conditions are associated with high health care resource use [1,2]. In older adults, mental conditions may be aggravated by concomitant medical health issues (eg, stroke), as many of them can no longer lead active lives because of cognitive and physical decline and psychosocial factors such as isolation and poverty. According to Statistics Canada, 5% of people aged ≥65 years perceive their mental health as fair or poor [3] and 6.3% of them have mood disorders [4]. Mental health disorders contribute significantly to morbidity and mortality in older adults and reduced quality of life. Regarding older adults with cognitive impairments such as dementia, up to 40%-50% of them experience depression at some point [5]. The high prevalence of mental health conditions in Canada places a substantial burden on individuals with mental illness, caregivers, and the health care system in general. For example, by 2015, the estimated public and private mental health expenditure was Can $15.8 (US $12.7) billion, representing 7% of the total health care expenditure, and by 2022 this expenditure is expected to increase by up to 9% [2]. Reminiscence therapy is a widely used nonpharmacological psychosocial intervention in people with mental health conditions and cognitive impairments such as dementia. Reminiscence therapy involves a discussion of past events and experiences, using tangible prompts to evoke memories or stimulate conversations [6]. The literature suggests that reminiscence therapy can significantly reduce social isolation, depression, and anxiety, while simultaneously improving quality of life in older people within urban aged care settings [6-8].

We believe that older adults can benefit significantly from information and telecommunication technologies as a strategy for improving mental health conditions such as depression and anxiety, while simultaneously improving their quality of life. Information and telecommunication technologies have the potential to improve the delivery of reminiscence therapy, as these technologies facilitate access to and the selection of biographical information and related contents, or by providing novel multimodal interaction forms (eg, virtual reality) to trigger memories [9,10]. Previous investigations have studied the impact of information and telecommunication technologies on reminiscence therapy on the quality of caregiver and patient relationships, subjective patients’ well-being [11], alleviating depressive symptoms in older adults [12], and supporting social interactions in residential care [13]. 3D immersive technologies (or immersive technologies) such as virtual reality and 3D display have also been shown to have a variety of positive effects on the mental, emotional, and social health of older adults [14]. In addition, there is an opportunity to indirectly reduce caregiver and health care professional burden by delivering stimulating viewing content to older adults affected by mental health conditions, thereby enhancing their daily experience. Although these immersive technologies are slowly becoming more accessible (ie, in terms of cost), there is a lack of literature on the benefits of 3D immersion technology for the mental health well-being of seniors. As a result, whether 3D immersion technology has a positive effect on older adults’ mental health conditions and the burden of self-care remains an open question.

3Scape Systems Inc is an Alberta-based private company that has produced a series of specialized 3D videos designed to simulate real-life events and engage individuals living with mental health disorders and cognitive impairments such as dementia [15]. These videos are based on the principles of reminiscence therapy, with each video highlighting a specific topic that is present in society, including animals, music, and nature. The goals of these videos are to trigger positive memories, engage individuals, and invite comfort and familiarity. Through a series of clinical evaluations, with the aim of determining the efficacy of the 3Scape system, qualitative and quantitative data were collected to ascertain whether the use of these immersive video productions has an impact on self-care or caregiver burden, the use of antipsychotic medications, mood, agitation, emotion, engagement, tolerability of 3D intervention, satisfaction with life, and the quality of life of older adults with and without cognitive impairment [16]. However, there were important threats to the internal validity of these studies, as there were no control conditions or groups; thus, the quality of evidence provided was very low.

### Evaluation Objectives and Research Questions

This study aims to explore the trial design and effect of 3Scape videos on older adults’ depressive and anxiety symptoms and quality of life and the efficacy in terms of caregiver burden of the Short-Term Assessment, Rehabilitation, and Treatment Psychiatry Day Hospital program at Glenrose Rehabilitation Hospital and to provide data to estimate the required parameters to design a definitive randomized controlled trial (RCT). Thus, this study has the following five research questions:

1. Is the designed protocol feasible for conducting a future definitive RCT?
2. Do the 3Scape videos affect older adults’ depressive and anxiety symptoms, mood, and overall quality of life compared with clients who receive the standard of care?
3. What are the overall experiences, beliefs, and attitudes of older adults while watching 3Scape videos?
4. What are the overall experiences, beliefs, and attitudes of therapists while watching 3Scape videos?
5. Do 3Scape videos affect caregivers’ burden compared with the same caregivers who provide the standard of care?

## Methods

### Study Design

**Figure 1:**
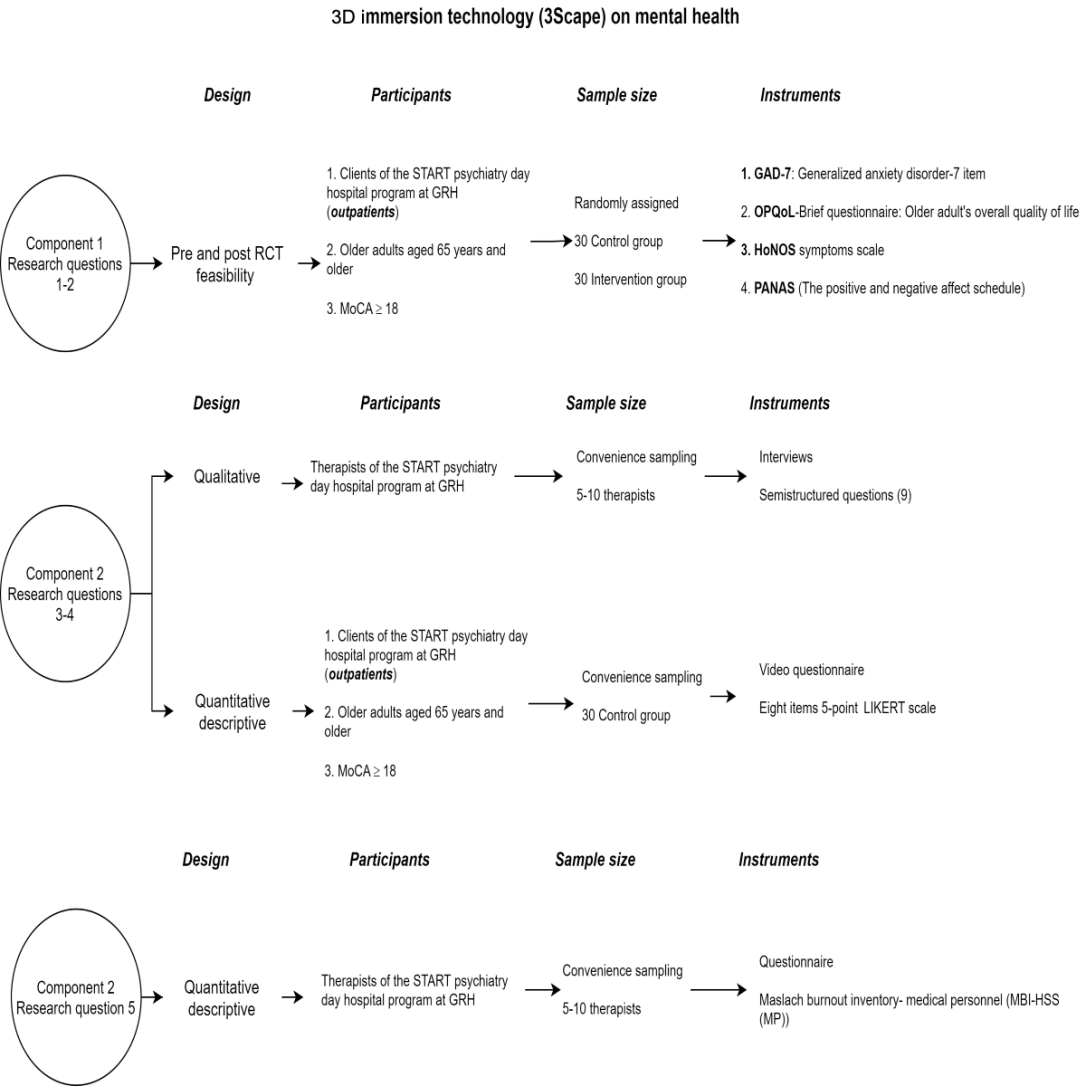
Study components. GRH: Glenrose Rehabilitation Hospital; HoNOS: Health of the Nation Outcome Scale; MoCA: Montreal Cognitive Assessment; OPQoL-Brief: Older People’s Quality of Life-Brief; RCT: randomized controlled trial.

This study will use a multimethod research design. The *Methods* section is presented in accordance with the study’s research questions. Additional details have been provided in Figure 1.

- Research questions 1-3, feasibility parallel-group RCT: The experimental group will receive the intervention, which consists of sessions with the 3Scape videos, whereas the control group will receive the standard of care used at the Short-Term Assessment, Rehabilitation, and Treatment Psychiatry Day Hospital program at Glenrose Rehabilitation Hospital (see the *Outcome Variables* section for more details). For this pilot study, we will follow the CONSORT (Consolidated Standards of Reporting Trials) guidelines for randomized feasibility studies [17]. The *Methods* section is presented with regard to the objectives of the study.
- Research question 4, study design: qualitative description design [18].
- Research question 5, study design: quantitative descriptive [19].

### Study Setting

This study will be conducted at the Short-Term Assessment, Rehabilitation, and Treatment Psychiatry Day Hospital at Glenrose Rehabilitation Hospital located in Edmonton, Alberta, Canada.

### Eligibility Criteria

#### Inclusion Criteria

The study participants will include adults aged ≥65 years attending the Short-Term Assessment, Rehabilitation, and Treatment Psychiatry Day Hospital program at Glenrose Rehabilitation Hospital who are cognitively intact or with minimal cognitive impairment; that is, Montreal Cognitive Assessment score ≥18 [20].

#### Exclusion Criteria

Potential participants who are unable to provide informed consent, are unable to communicate in the English language, have a significant sensory impairment (which would prevent adequate viewing of the 3D videos), have a mental health diagnosis with behavioral disturbances such as the potential for aggression or severe agitation, with aphasia, or other diagnoses that would prevent the participant from completing surveys, or have posttraumatic stress disorder or zoophobia will be excluded.

### Interventions

The eligible participants will be randomly assigned 1:1 to either the experimental group or the control group.

#### Control Group

This group will receive standard care consisting of (1) standard psychiatric nursing care (including some individual counseling), (2) medication trials or titration (antidepressants, antianxiety medicines, sleep medicines), and (3) group therapies (psychoeducation, cognitive behavioral therapy, stress management groups, dialectical behavior therapy, exercise groups, community engagement and wellness groups, leisure groups, and grief and loss groups). Every group runs twice a week for 6 weeks (1.5 months). The patients simultaneously attend 3 therapy groups for 6 weeks. That is, the patients attend 3 therapy sessions per day, twice a week. Each patient attends every group at some point during their 20-week stay in the program. Each group session is 1 hour long. Therefore, the patients receive 36 hours of standard care every 6 weeks for a total of 108 hours on the Short-Term Assessment, Rehabilitation, and Treatment program.

#### Experimental (Intervention) Group

In the experimental arm, the psychoeducation (Leisure Choices) group therapy (on the Short-Term Assessment, Rehabilitation, and Treatment program) will be replaced by five 3D video screenings (the intervention); in addition, they will receive the same standard care. The five 3D video screening sessions will be delivered as one session per week within a 6-week time frame. Each session will take approximately 1 hour, consisting of one 20-minute video screening and a postvideo screening discussion and questionnaire. A therapist and a member of the research team will be present during the video screening sessions. The therapist will lead the video screening sessions and the postvideo screening discussion (20 minutes of video and 20 minutes of discussion). The research team will observe alertness during video screenings sessions. After the discussion, the research team will administer two questionnaires (Positive and Negative Affect Schedule [PANAS] and postscreening survey), which will take approximately 20 minutes. Each session will take 1 hour overall. The videos will be screened in any order. The goal of these videos is to trigger positive memories, engage individuals, and bring comfort and familiarity. The topics of the videos are as follows: (1) *The Path*, (2) *Remembering*, (3) *The Dance*, (4) *The Memory Box*, and (5) *Baby Animals*. A sample of the videos can be found on the company’s website [15]. The sessions will take place at the Courage Center at Glenrose Rehabilitation Hospital. No more than 5 participants will be present during each video screening session.

### Outcome Variables

#### Research Questions 1-2 (Primary Outcome Variables)

Older adults’ depressive and anxiety symptoms, mood, overall quality of life, and engagement are the primary outcome variables for these research questions. Older adults’ depressive and anxiety symptoms will be assessed using the Generalized Anxiety Disorder-7 item (GAD-7) scale patient self-report tool [21] and the Health of the Nation Outcome Scale (HoNOS; a clinician rating tool) symptoms [22], older adults’ mood will be measured using PANAS (20-item self-report questionnaire) [23], and older adults’ overall quality of life will be measured using Older People’s Quality of Life-Brief (OPQoL-Brief) questionnaire scale-13 items self-reported tool [24].

#### Research Questions 1-2 (Secondary Outcome Variables)

Engagement while watching the 3Scape videos will be measured using an engagement scale developed by team members in a previous study [25]. This is an 8-point Likert scale that has been shown to be understood by older adults, even those with mild cognitive impairments.

#### Research Question 3 (Participants: Older Adults)

Participants will be asked to participate in a postsurvey to provide feedback on their experiences while watching the 3Scape videos. The overall experience, beliefs, and attitudes of older adults while watching the 3Scape videos will be measured using an instrument (height items 5-point Likert scale and 4 open-ended questions) developed by the University of Calgary in a previous study on this technology.

#### Research Question 4 (Participants: Therapists)

Participants will be asked to participate in a poststudy interview to provide feedback on their experiences, with their patients using the 3Scape videos*.*

#### Research Question 5 (Participants: Therapists)

The caregiver burden will be assessed by using the burnout and workload perception among health care providers, measured by the Maslach Burnout Inventory-Human Services Survey for Medical Personnel (MBI-HSS MP) [26].

All of the measures have good to excellent metric properties [27,28].

### Independent Variable

The independent variable in this study is the type of intervention used. This variable has two levels (ie, control and intervention); the *Interventions* section provides more details.

### Confounding Variables

Age, gender, mental health condition, and groups in which the patients are involved during the intervention will be applied, and whether the patients are taking any medication will be analyzed as confounding variables.

### Participant Timeline

#### Overview

**Figure 2:**
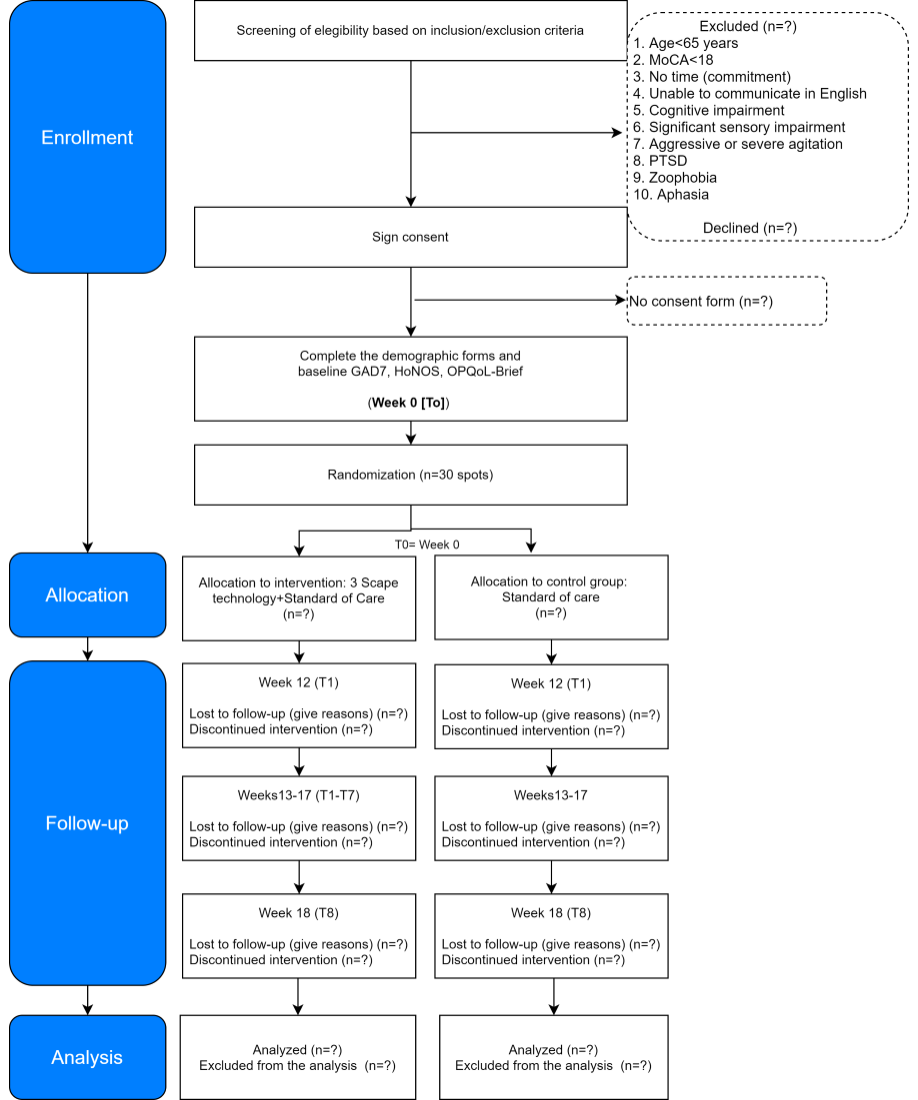
Flow of participants. GAD-7: Generalized Anxiety Disorder-7 item; HoNOS: Health of the Nation Outcome Scale; MoCA: Montreal Cognitive Assessment; OPQoL-Brief: Older People’s Quality of Life-Brief; PTSD: posttraumatic stress disorder.

**Figure 3:**
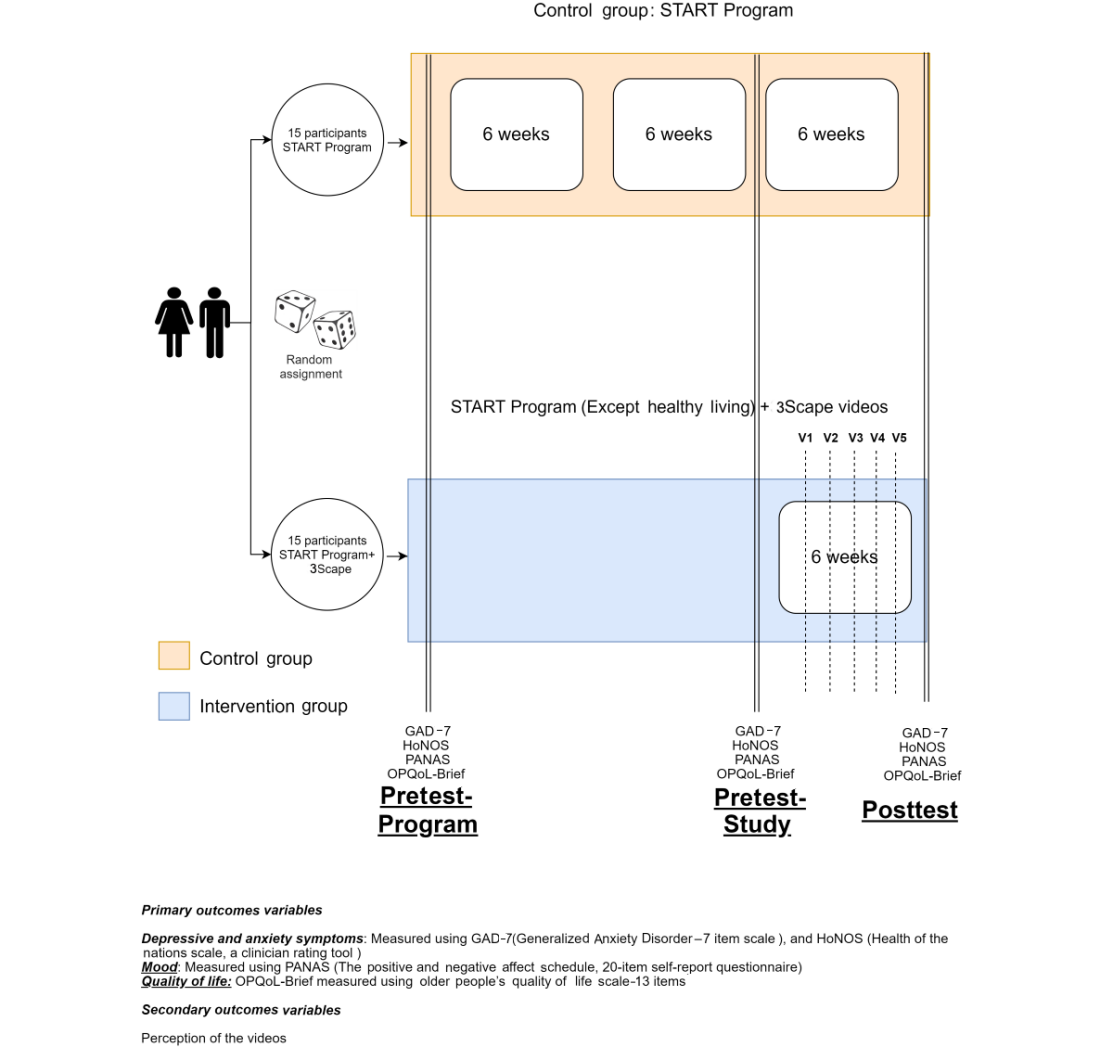
Study design schema.

This feasibility trial consisted of a 6-week intervention treatment phase; this study does not have a follow-up phase. The total trial data collection period will be 8 months. As shown in Table 1, Figure 2, and Figure 3, the measurements will be taken as follows.

**Table 1:** Key variables and measurements.

Variables			Participants		Measurement		T_0_		T_1_		T_2_		T_3_		T_5_		T_6_		T_7_		T_8_
**Primary outcome**																					
	GAD-7^a^	Older adults		GAD-7 scale		✓^b,c,d^		✓^c,d^		✓^d^		✓^d^		✓^d^		✓^d^		✓^d^		✓^c,d^	
	HoNOS^e^	Older adults		HoNOS, a clinician rating tool		✓^c,d^		✓^c,d^		✓^d^		✓^d^		✓^d^		✓^d^		✓^d^		✓^c,d^	
	OPQoL-Brief^f^	Older adults		OPQoL-Brief scale		✓^c,d^		✓^c,d^		✓^d^		✓^d^		✓^d^		✓^d^		✓^d^		✓^c,d^	
	PANAS^g^	Older adults		PANAS		✓^c,d^		✓^c,d^		✓^d^		✓^d^		✓^d^		✓^d^		✓^d^		✓^c,d^	
**Secondary outcomes**																					
	Engagement	Older adults		Engagement scale		✓^d^		✓^d^		✓^d^		✓^d^		✓^d^		✓^d^		✓^d^		✓^d^	
	Experiences while watching the 3Scape videos	Older adults and therapists		Semistructured interview																✓	
	MBI-HSS MP^h^	Therapists		MBI-HSS MP																✓	
**Covariates**																					
	Demographics	Older adults and therapists		Self-report assessment questionnaire		✓^c,d^															

^a^GAD-7: Generalized Anxiety Disorder-7.

^b^Outcome present.

^c^Control groups.

^d^Intervention groups.

^e^HoNOS: Health of the Nation Outcome Scale.

^f^OPQoL-Brief: Older People’s Quality of Life-Brief.

^g^PANAS: Positive and Negative Affect Schedule.

^h^MBI-HSS MP: Maslach Burnout Inventory-Human Services Survey for Medical Personnel.

#### Intervention Group (Participants: Older Adults)

Research questions 1-2 (primary outcome variables): at the enrollment of the program (week 0 [T_0_], called the pretest program enrollment), the pretest before starting showing the videos (week 12 [T_1_]), and at the posttest when the participants finish the program and watch the videos (week 18 [T_8_]).

- Research questions 1-2 (secondary outcome variables): after watching each video, that is, weeks 13-17 (T_1-7_).
- Research question 3 (participants, older adults): after watching every video (week 18 [T_8_]).

#### Control Group (Participants: Older Adults)

- Research question 1-2 (Primary outcome variables): at the enrollment of the program (week 0 [T_0_], called the pretest program enrollment), week 12 (T_1_), and at the posttest when the participants finish the program and watch the videos (week 18 [T_8_]).

For the therapists involved in the intervention group, the measurements will be taken as follows: research questions 4 and 5 (participants, therapists): week 12 [T_1_].

### Sample Size

#### Research Questions 1-3 (Participants: Older Adults)

As this is a feasibility study, a sample size calculation is not required [17]. However, we can estimate the number of participants we will be able to recruit during the data collection period. In this feasibility study with a statistical power of 0.8, an α of .05, and an effect size of 1.20, the minimum required sample size will be 24 participants in total (12 in each group [19]). We aim to recruit 15 participants for each group, for a total sample size of 30, to compensate for a 20% dropout rate. Sample size calculations are estimated using G*Power (version 3.1.9.4, Universitat Kiel) [29].

#### Research Questions 4-5 (Participants: Therapists)

We aim to recruit 5-10 therapists participating in the Short-Term Assessment, Rehabilitation, and Treatment Psychiatry Day Hospital program who provided the intervention.

### Recruitment

#### Participants (Older Adults)

An invitation to participate will be posted in the Short-Term Assessment, Rehabilitation, and Treatment Psychiatry Day Hospital program at Glenrose Rehabilitation Hospital. Therapists will support recruitment strategies, including the provision of information sessions and one-on-one conversations with potential participants. The first contact with a potential participant will be made through one of the therapists not involved in the research team. Therapists who are already involved in the clinical care of the patients will then determine the individuals’ willingness to be approached by the therapist researcher regarding participation and then obtain their consent for the study, as the case may be.

#### Allocation

##### Sequence Generation

Probability sampling will be used. Random sequence generation will be prepared in advance by a research team member (AMC) on an Excel file spreadsheet (RAND function) using permuted block randomization with a block size of 4 and a ratio of 1:1.

##### Concealment Mechanism

Allocation concealment will be ensured, as we will not release the randomization code until the patients have been recruited to the trial and all of the baseline measurements have been completed.

##### Implementation

If a potential participant meets the inclusion criteria, the therapist researcher (MKW) will ask the study coordinator to check whether a place is available in the study for that participant in a given group. If a place is available, then the therapist researcher (MKW), or another therapist involved in the recruitment process, will invite the participant to participate in the study, explain the study to him or her, and ask him or her to sign the consent form. If a potential participant is assigned to a particular therapist researcher (MKW), the therapist would not invite the participant to participate in the study. Instead, a secondary therapist researcher will do so. As a result, the freedom to decline will not be compromised. Once the participants or their substitute decision makers have signed the consent form and given their assent, the therapist researcher (MKW) will inform the study coordinator. The study coordinator will allocate each participant to 1 arm of the trial according to the allocation protocol and will assign a code. This code will be given to the therapist researcher (MKW) and research assistants (RAs) who will conduct the assessments.

#### Blinding (Masking)

The assessments of outcome variables will be conducted by RAs who are blinded to the treatment allocation. Owing to the nature of the intervention, neither the participants nor the therapist can be blinded to the treatment allocation, but they are strongly encouraged not to disclose the participants’ allocation status during the assessments. An RA will enter the data into a computer on separate datasheets, and a senior RA will conduct the data analysis under the supervision of the principal investigator (PI; AMC).

### Data Collection Methods

#### Research Questions 1-2 (Participants: Older Adults)

A therapist on the Short-Term Assessment, Rehabilitation, and Treatment Psychiatry Day Hospital program at Glenrose Rehabilitation Hospital with an RA, that is, RA 1, will administer the intervention (5 video screenings). The control group will receive standard care at the Short-Term Assessment, Rehabilitation, and Treatment Psychiatry Day Hospital program Glenrose Rehabilitation Hospital. The PANAS scale will be administered after each session along with the 3Scape videos to the intervention group participants (10 minutes). The GAD-7, HoNOS, and OPQoL-Brief will be administered to all participants at the beginning and end of the Short-Term Assessment, Rehabilitation, and Treatment program, as this is part of the standard care. In addition, we will administer the same measures (ie, GAD-7, HoNOS, and OPQoL-Brief) to the participants on both arms before and after each group therapy is conducted (ie, 6 weeks apart). The administration of these measures will take approximately 30 minutes. RA 2, who will be blinded to the group allocation, will administer these measures. The participants will be asked not to inform the evaluators about the kind of intervention they had received. The sessions will be conducted in a quiet room at the Courage Center.

#### Research Question 3 (Participants: Older Adults)

The overall experience, beliefs, and attitudes of older adults while watching the 3Scape videos will be measured using an instrument (8 items on a 5-point Likert scale and 4 open-ended questions) developed by the University of Calgary in a previous study on this technology.

#### Research Question 4

Interviews will be conducted for the therapists. Semistructured questions (topic guided) will examine the usefulness of 3Scape videos for the treatment of the mental health condition of older adults. Interviews will be audiotaped for later analysis by team members. To ensure anonymity, the older adults’ and therapists’ responses will not be connected to their identities. RA 2 will conduct the surveys. The administration of these measures will take approximately 15 minutes.

#### Research Question 5

The MBI-HSS MP will be administered to every therapist who provides the intervention to study the participants at the pretest and posttest. The administration of these measures will take approximately 20-30 minutes. RA 2 will administer these measures.

### Data Analyses

#### Research Questions 1, 2, and 3

The analyses will be conducted in SPSS using intention-to-treat principles. Descriptive statistics will be used to characterize the groups during the pretest. Owing to the small sample size, comparisons of the GAD-7, HoNOS, OPQoL-Brief, and PANAS scores within the groups will be performed using a Wilcoxon signed-rank test (*P*≤.05), and comparisons between the groups will be calculated using the Mann–Whitney *U* test (*P*≤.05). The GAD-7, HoNOS, OPQoL-Brief, and PANAS scores will be used to analyze the clinical significance, established as a change of 2 or more units [30]. Participants’ engagement will be analyzed using descriptive statistics.

#### Research Question 4

The audiotapes will be transcribed and content analysis will be performed. The content analysis will be data driven. Data codes will be inductively generated using the data collected. Through the coding, a small number of themes or categories will be generated. The analyses will be performed by an RA, and a consensus in the interpretations will be achieved through a discussion among the research team members. The validity of the interpretations will be discussed with and agreed upon by every member of the research team. NVivo 10 software will be used to conduct data analysis.

#### Research Question 5

Descriptive statistics will be used to characterize the groups at pretest and posttest. Owing to the small sample size, comparisons of the MBI-HSS MP scores within the groups will be performed using a Wilcoxon signed-rank test (*P*≤.05), and comparisons between the groups will be calculated using a Mann–Whitney *U* test (*P*≤.05).

### Ethics and Dissemination

#### Research Ethics Approval

All procedures were approved by the ethics committee of Alberta University and the Northern Alberta Clinical Trials Research Centre, Canada.

#### Incentives

The participants will not receive any incentive for participating in this study.

#### Withdrawal From the Study

The participants and substitute decision makers can request to withdraw from the study at any time either orally or in writing. The participants will be able to withdraw from the study at any time before the group analysis is calculated. If a participant withdraws, his or her information will not be taken into account in the analysis. In the event that a participant requests to have his or her data destroyed, the research team will honor this request by shredding and recycling the paper records and erasing any records stored on a computer hard drive using commercial software applications designed to remove all data from storage devices. However, once all of the participants’ data have been analyzed, the participant cannot withdraw. The participants will be informed of this in a consent letter. The deadline for withdrawal will be once all of the participants’ data have been collected and data analysis is underway. This will occur during the 8 months of the study.

#### Consent or Assent

Signed consent will be obtained from all participants in the study. For those who are unable to provide their informed consent, one of the therapist researcher (MKW), or another therapist involved in the recruitment process, will approach each potential participant and his or her substitute decision maker to provide information on the study. If these potential participants and their substitute decision makers provide their consent, the substitute decision makers will sign the consent form, and we will seek the potential participants’ assent.

#### Confidentiality

We will assign numerical codes to the participants instead of using their names or other identifiers. Only the study coordinator will have access to the master list, where these codes are linked to the participants’ first names. With the exception of direct conversations with each participant, their names will not be used, only their numbers. Hard copies of the consent forms, questionnaires, and study notes will be stored in a locked filing cabinet in a laboratory (Corbett Hall 1-45, Faculty of Rehabilitation Medicine, University of Alberta). All of the de-identified electronic study documents will be encrypted and stored on a password-protected computer located in a laboratory (Corbett Hall 1-45, Faculty of Rehabilitation Medicine, University of Alberta).

#### Access to Data

All PIs will be given access to the cleaned data sets. The master list will be stored on a password-protected computer, located in the PI’s laboratory (Corbett Hall 1-45, Faculty of Rehabilitation Medicine, University of Alberta). Only the study coordinator will have access to the master list. The data will be retained for 5 years. There are no plans for future use of data other than publishing them in peer-reviewed journals and at conferences. The data will not become part of a data repository and will not be involved in the creation of a research database or registry for future research use. After 5 years, the data will be destroyed. This will be done by shredding the paper records. Records stored on a computer hard drive will be erased using commercial software applications designed to remove all data from storage devices.

#### Quality Assurance and Safety

We will follow the CONSORT guidelines for clinical trial feasibility [17]. In addition, we will assess the quality of our study using the Physiotherapy Evidence Database scale [19].

## Results

The 3Scape study was launched in April 2020. As of August 2021, our study is on hold due to the COVID-19 pandemic. The recruitment process is expected to resume by November 2021, and the primary impact analysis is expected to be conducted by February 2022. This project is an excellent example of how industry and the health care system can support each other to grow and diversify Alberta’s economy, and promote the entry of this valuable technology into the global rehabilitation market.

## Discussion

### Principal Findings

The primary objective of this proposed study is to assess the feasibility of conducting a definitive trial on the effectiveness of 3Scape technologies. The results of this project will inform the development of best practices for older adults with mental health conditions such as depression and anxiety, which affect as much as 6.3% of the population of older adults. Currently, our study is on hold because of the COVID-19 pandemic. The recruitment process is expected to resume by November 2020, and the primary impact analysis is expected to be conducted by February 2021.

The level of evidence that immersive technologies have an impact on older adults’ mental health conditions and the burden of care is low. The 3Scape Systems Inc videos have the potential to become a sound alternative at the midpoint between these 2 extremes. 3Scape Systems has a technological readiness level of 9 [31], thus having a sufficient level of readiness that can be tested in a real-world clinical setting. This feasibility study is the first RCT to evaluate the potential benefits of 3Scape Systems. Conducting this RCT will provide valuable information. First, the estimates can be used for sample size calculations in future RCTs. Second, as we will measure the patients’ outcome variables on 4 different occasions during the intervention, the results of this study will guide therapists on the expected percentage of a patient’s improvement and how progress was achieved over a period of 10 weeks. The results of this project will provide them with information on the feasibility of adopting 3Scape Systems.

### Conclusions

In conclusion, this study will provide valuable information such as the measurement of comparative intervention effects, perception of older adults and therapists about the 3Scape Systems, the associated costs of treatment, and product costings. This will contribute to the evidence planning process, which will be crucial for the future adoption of 3Scape Systems.

## Abbreviations

CONSORT: Consolidated Standards of Reporting Trials
GAD-7: Generalized Anxiety Disorder-7 item
MBI-HSS MP: Maslach Burnout Inventory-Human Services Survey for Medical Personnel
OPQoL-Brief: Older People’s Quality of Life-Brief
PANAS: Positive and Negative Affect Schedule
PI: principal investigator
RA: research assistant
RCT: randomized controlled trial
